# Relationship between the oospore dose in the leaf litter above the vineyard ground and primary infections by *Plasmopara viticola*

**DOI:** 10.3389/fpls.2025.1524959

**Published:** 2025-03-07

**Authors:** Giorgia Fedele, Giuliana Maddalena, Margherita Furiosi, Vittorio Rossi, Silvia Laura Toffolatti, Tito Caffi

**Affiliations:** ^1^ Department of Sustainable Crop Production, Università Cattolica dal Sacro Cuore, Piacenza, Italy; ^2^ Research Center on Plant Health Modelling, Department of Sustainable Crop Production, Università Cattolica dal Sacro Cuore, Piacenza, Italy; ^3^ Dipartimento di Scienze Agrarie e Ambientali, Università degli Studi di Milano, Milan, Italy

**Keywords:** *Vitis vinifera*, sexual stage, primary inoculum quantification, duplex qPCR, oospore germination

## Abstract

*Plasmopara viticola*, the grapevine downy mildew (DM) oomycete, overwinters as oospores in fallen leaves above the vineyard ground. The oospores repeatedly germinate in the following season, causing primary infections on the leaves and clusters. In the present study, the relationship between the numbers of *P. viticola* oospores in the leaf litter and the dynamics of primary infections on grape leaves were studied for three years to assess whether the assessment of the oospore pool in a vineyard can provide information on the DM pressure. Five leaf litters were prepared by mixing DM-free and -affected leaves in varying proportions in the fall, including 100% DM-free leaves (LL0), 75% DM-free and 25% DM-affected leaves (LL25), 50% DM-free and 50% DM-affected leaves (LL50), 25% DM-free and 75% DM-affected leaves (LL75), and 100% DM-affected leaves (LL100). The leaf litters were overwintered in a vineyard and the oospore pool was quantified in the following season by counting the oospore numbers and assessing *P. viticola* DNA (*Pv* DNA) through quantitative polymerase chain reaction (qPCR). There were significant correlations between the prevalence of DM-affected leaves in the leaf litter, the number of oospores (r = 0.969), and the molecular infestation index (MII) calculated based on *Pv* DNA (r = 0.974). In addition, there were significant correlations between oospore numbers and MII (r = 0.895). Survival analysis showed a significant effect of diseased leaves in the leaf litter on relevant DM onset time during the primary inoculum season. DM lesions on plants increased with an increasing proportion of DM-affected leaves in the leaf litter, with plants above LL100 exhibiting four-fold more lesions than the plants above LL0. Results show that there is a legacy/inheritance factor in a vineyard, which is linked to the oospore pool. This is a crucial factor influencing the initial onset and severity of the disease and thus the level of alert for achieving good DM control in the early season. The qPCR assay can be used to assess the legacy/inheritance factor and inform early-season disease control. This data could be used to devise an effective strategy for leaf residue and soil management in vineyards.

## 1 Introduction

Grapevine downy mildew (DM) is caused by the oomycete *Plasmopara viticola* (Berk et Curt.) Berlese and de Toni. This organism severely affects the vineyards located in areas with frequent rain and planted with susceptible cultivars of *Vitis vinifera* (Lafon and Clerjeau, 1988).

*Plasmopara viticola* has dimorphic reproductive spores, i.e., sexual and asexual spores, responsible for primary and secondary DM infections, respectively. The oospores represent the sexual stage of *P.viticola* and are formed after the heterothallic fusion of an antheridium and an oogonium coming from two compatible mating types in the affected leaf tissue from mid-summer to autumn (Burruano, 2000; Wong et al., 2001; Scherer and Gisi, 2006). Oospore formation occurs under a wide range of temperatures during leaf senescence; however, it seems to be favored under dry conditions (Rouzet and Jacquin, 2003; Gessler et al., 2011). Oospores are generally produced in large numbers, easily reaching 250 oospores/mm^2^ in a polygonal fleck (Gessler et al., 2011). Their formation is usually delimited by the veins of a leaf that exhibit a mosaic symptom (Gessler et al., 2011). Oospores overwinter in the leaf litter above the soil surface (Kennelly et al., 2007; Rossi et al., 2013) and, following leaf residue degradation, in the soil (Yang et al., 2023). In wintertime, oospores reach morphological maturity; their walls become thick, their nuclei fuse, an ooplast is formed, and large lipid globules break down into smaller ones (Vercesi et al., 1999). Prompt germination of morphologically mature oospores is, however, prevented by dormancy (Galet, 1977), a process regulated by the environment, nutrient permeability, and endogenous inhibitors. At the end of their dormancy, with a phenotypic synchrony with the host plant (Maddalena et al., 2021), oospores germinate under favorable environmental conditions (Rossi and Caffi, 2007). Oospore germination ends with the formation of a macrosporangium containing zoospores (Galbiati and Longhin, 1984). The germination of oospores requires minimal and optimal temperatures of 12–13°C and 20–24°C, respectively (Gessler et al., 2011; Laviola et al., 1986). Oospore germination also requires moistening the leaf litter by rainfall or water flux from the atmosphere (Arens, 1929; Rossi et al., 2008a; Rossi et al., 2013). Dry conditions prolong the dormant period of the oospores, potentially damaging them and preventing germination (Arens, 1929; Gessler et al., 2011; Vercesi et al., 2010). Some oospores can remain dormant but viable for an entire season or even for some years (Kennelly et al., 2007; Caffi et al., 2011).

Oospores are the sole source of the primary inoculum of *P. viticola* and were long considered to trigger the epidemic in the early grapevine season, with subsequent increase in the disease severity being attributed to asexual multiplication and secondary infections (Blaeser and Weltzien, 1979; Lalancette et al., 1988; Lafon and Clerjeau, 1988). The use of DNA microsatellites, which enables the identification of genotypes causing a single DM leaf lesion, showed that new *P. viticola* genotypes enter the epidemic during most of the grape-growing season. This finding indicated that oospores continue to germinate throughout the season and the primary inoculum not only triggers epidemics but contributes to their progress (Kump et al., 1998; Gobbin et al., 2003; Rumbou and Gessler, 2004; Gobbin et al., 2005; Gobbin et al., 2006). The oospore pool of a vineyard then influences the epidemic in the following season. Carisse (2016) reported that a large number of leaves affected by DM in the fall (which would result in a large number of oospores) were associated with an earlier disease onset and highly severe disease in the following spring. However, a clear relationship between the prevalence of affected leaves in the fall, the numbers of oospores, and primary lesions in the next season has not yet been established.

The present work aimed to investigate the relationship between the number of *P. viticola* oospores that overwintered in the leaf litter above the vineyard ground and the dynamics of primary infections on grape leaves. For this purpose, we collected both DM-free and -affected grape leaves before leaf fall in a season and mixed them in different proportions to create five different leaf litters. These leaf litters were overwintered above the vineyard soil and the oospore pool was quantified at the bud break of vines in the following season. Both oospore numbers and *P. viticola* DNA (*Pv* DNA) were quantified at this stage. The onset of DM lesions on leaves was observed during the primary inoculum season (i.e., the season in which the inoculum generated by oospores contributes to epidemic development; Caffi et al., 2009) to verify whether the disease onset and severity differed for different leaf litters and oospore pools. The final goal was to verify whether assessing the oospore pool in a vineyard before the growing season of vines can provide information on the DM pressure in the vineyard, to be used for informing disease management.

## 2 Materials and methods

### 2.1 Leaf litter preparation and management

Grapevine leaves showing typical, mosaic-like DM symptoms (hereinafter referred to as DM- affected) were collected from September to October in 2020, 2021, and 2022 from untreated plots in different vineyards located across northern Italy to collect an oospore population not subjected to fungicide treatments to limit any possible effects of fungicide treatments on oospores viability. Leaves with no DM symptoms (hereinafter referred to as DM-free) were also collected. Although still attached to the vines at the time of collection, the DM-affected leaves were diseased and senescent and would have soon fallen to the soil surface, constituting the vineyard leaf litter with oospores naturally formed and matured.

Leaves were transported to the laboratory, where the presence of *P. viticola* oospores in the DM- affected leaves was confirmed. A random sample of approximately 100 leaf pieces (1–2 cm^2^) with lesions were immersed in an acetic acid-ethanol (1:3 *v/v*) solution overnight at room temperature. The bleached leaf pieces were rinsed in distilled water and examined under a light microscope at 40–80x magnification.

The leaves were then spread on an absorbent paper in a thin layer and incubated at room temperature (22–24°C) for drying. The DM-affected and -free leaves were kept separate. The leaves were then crushed manually into small pieces (2–4 cm wide). The leaf pieces of the two groups (DM-affected and -free) were then mixed to form leaf litters with the following proportions (based on the leaves weight): I) 100% DM-free (hereinafter named LL0), II) 75% DM-free and 25% DM-affected (LL25), III) 50% DM-free and 50% DM-affected (LL50), IV) 25% DM-free and 75% DM-affected (LL75), and V) 100 DM-affected (LL100). The five leaf litters were then considered to hold an increasing number of *P. viticola* oospores.

### 2.2 Experimental vineyard

**Figure 1:**
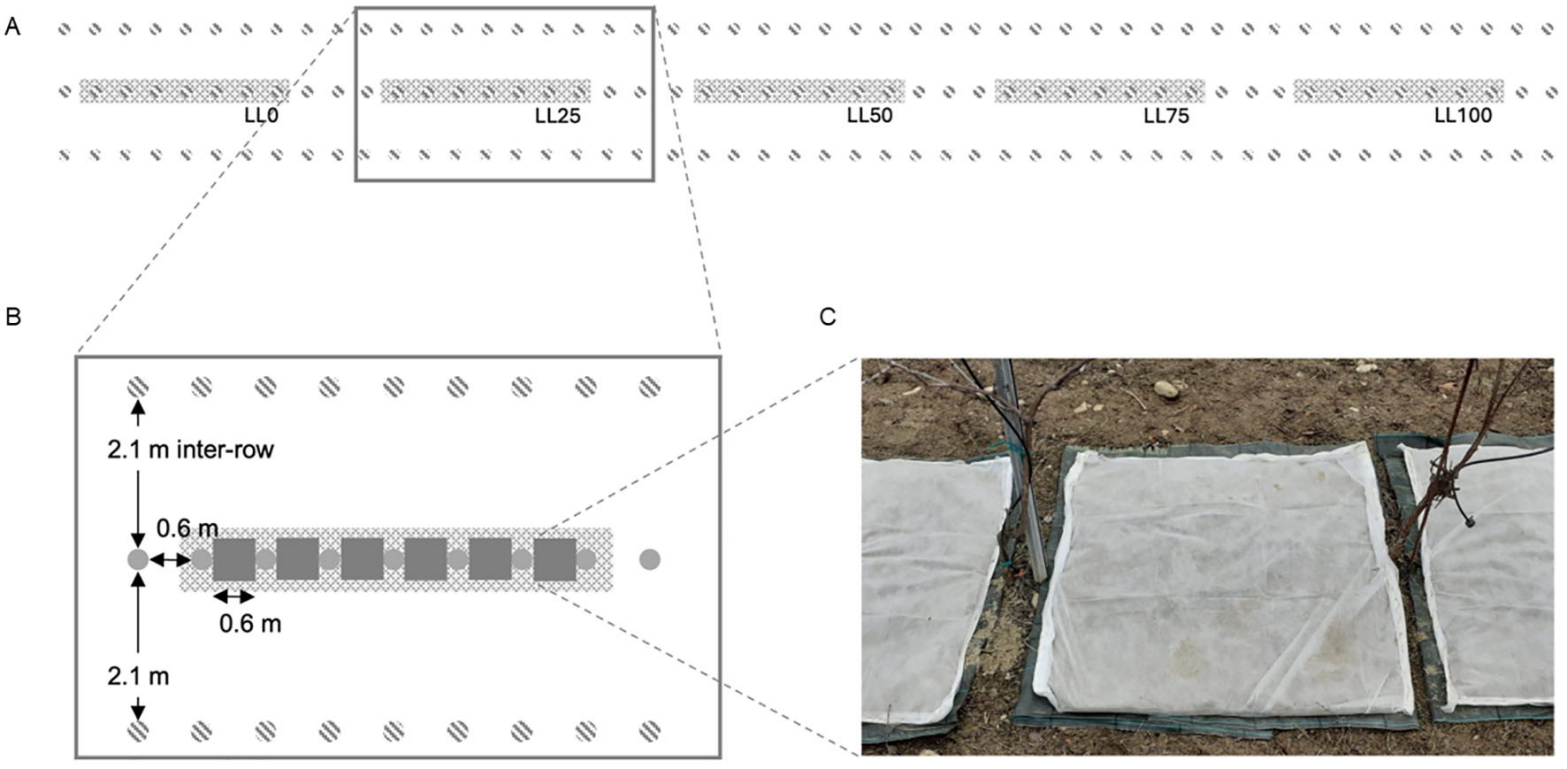
**(A)** Experimental setting showing five blocks (grey areas), each of them comprising seven contiguous plants (separated by three plants), arranged in a row of grapevine cv. Merlot (grey, full dots in the central row), surrounded by two rows of downy mildew (DM)-resistant varieties (grey, dashed dots); the first row of DM-resistant varieties bordered a wide grassy area while the second row bordered other rows of cv. Merlot, which were treated with fungicides to prevent downy mildew on a calendar basis; **(B)** the plants were spaced 0.6 m along the row, and the rows were 2.1 m apart; leaf litters (grey squares) were located between contiguous plants covering a surface of 0.36 m^2^; and **(C)** leaf litter setting composed by a layer of plastic mesh (1 cm mesh), a non-woven fabric cloth, leaf pieces, a wire mesh (2 cm mesh), and a second non-woven fabric cloth.

The study was conducted in an experimental vineyard in the University campus at Piacenza, Northern Italy (45°2’5” N, 9°43’46” E). The vineyard (700 m^2^) was 2-years old in 2020 and planted with cv. Merlot and DM-resistant varieties. The plants were spaced 2.1 m in inter-row and 0.6 m apart along the row. They were trained with a single-cordon Guyot system, with eight buds per cane. The vineyard was managed as per the common practice. The soil in the rows and inter-rows was bare. A row of Merlot (on a planting area of 63 m^2^), located between two rows of DM-resistant varieties, was used and split into two plots (replicates). Each plot was, in turn, split into five blocks. Each block was composed by seven contiguous plants and separated to the adjacent block by three plants (**Figure 1**); no fungicides were used on these plants until the end of the experiment. The experiment was repeated each year on the same plots following the same experimental design. At the end of the leaf fall, the soil of the inter-row and along the row in each block was first carefully cleared of the leaves that fell to the ground. The ground (0.6×0.6 m) between two contiguous plants was covered with a layer of plastic mesh (1 cm mesh) and with a non-woven fabric cloth, both fixed to the ground with pegs. Leaf pieces (200 g) were arranged over the cloth to form a 2–4 cm thick layer and covered with a wire mesh (2 cm mesh) and a second non-woven fabric cloth, both also fixed to the ground. The aim was to create a leaf litter overwintering on the vineyard ground, avoiding its dispersal by wind or animals or loss in soil. The five leaf litters were randomly arranged in the two plots. At bud-break, the top layer of non-woven fabric cloth was removed to avoid any potential interference with the splashing droplets originated by the raindrop impacted on the leaf litter.

The temperature (T, °C), relative humidity (RH, %), precipitation (mm), leaf wetness (LW, yes/no), and wind speed (m/s) were recorded every hour by a weather station (iMetos^®^, Pessl Instruments) located in the experimental vineyard for the entire duration of the experiment.

### 2.3 Quantification of oospores in leaf litters

Two quantification methods were used: microscope counts and quantitative polymerase chain reaction (qPCR). At the beginning of each sampling season (mid-March), four aliquots of 1 g of leaf litter were collected from each leaf litter replicate; 500 mg were used for microscope counts and 500 mg for qPCR.

#### 2.3.1 Microscopy

For microscope counts, oospore suspensions were obtained from leaf litter samples as previously described by Vercesi et al. (2000), with a few modifications. Briefly, the leaf pieces were weighed with an analytical balance (RADWAG PS45000/C2. Radom, Poland) and finely ground in a glass Potter tissue grinder containing sterile-distilled water. The homogenate was double-filtered using 100-μm pore size filters to remove larger leaf debris, followed by thorough washing with sterile- distilled water through 45-μm filters. The material retained on the 45-μm mesh filter, presumed to contain the oospores based on their size, was resuspended in sterile distilled water. The concentration of oospores was determined by counting the number of oospores present in two 10-μL drops of the suspension under a stereomicroscope. The counts were averaged, and the total number of oospores in the entire suspension, whose volume was known, was calculated. The counts were expressed as the number of oospores per g of leaf litter. The latter weight refers to the weight of dry leaves obtained after drying at 65°C for 24 h.

#### 2.3.2 qPCR

Each leaf litter sample was placed on a sieve or filter mesh and thoroughly washed with water to remove soil dirt. Then, each sample was dried using a damping paper first. Then, the sample was put under a laminar flow and left to dry out for 1 h (Si Ammour et al., 2020). Each sample was divided into four biological replicates of 100 mg each and finally stored in a freezer (−20°C) until DNA extraction.

##### 2.3.2.1 DNA extraction

Genomic DNA was extracted from leaf litter samples, as previously described by Taibi et al. (2023). Briefly, the leaf pieces were put in a mortar, freeze-dried with liquid nitrogen, and ground into a fine powder that was inserted in sterile, safe-lock 2-mL tubes (Eppendorf AG, Germany). Then, 100–150 mg of the leaf sample powder was placed in a 2-mL microcentrifuge tube containing 650 μL of cetyltrimethylammonium bromide (CTAB) extraction buffer (2% CTAB, 100 mM Tris-HCl (pH 8.0), 20 mM ethylenediaminetetraacetic acid [EDTA], 1.4 M NaCl, and 1% polyvinylpyrrolidone [PVP]), 100 mg of glass sand (425–600 μm in diameter), and two stainless steel beads (5 mm in diameter; Qiagen, Italy). All tubes were then placed in a TissueLyser II (Qiagen, Italy), shaken for 1 min at 30 cycles/s, and placed in a heat block at 65°C for 90 min. Total DNA was purified with chloroform-isoamyl alcohol (24:1, *v/v*), precipitated with ice-cooled isopropanol, washed with 70% ethanol, and resuspended in 40 μL of sterile ultrapure water. The yield and purity of the extracted DNA were determined using NanoPhotometer^®^ N60 (Implen GmbH, Germany). The total concentration of the DNA from each sample was finally adjusted to 20 ng/μL.

##### 2.3.2.2 Primers and hydrolysis probes

To quantify the *Pv* DNA, we used two specific primers and a hydrolysis probe (Giop) designed to target the internal transcribed spacer 1 (ITS 1)-5.8S rDNA. The *V. vinifera* DNA was also quantified to normalize the *P. viticola* quantification in plant tissues (Valsesia et al., 2005). Two specific primers and a hydrolysis probe (Res) designed to target resveratrol synthase gene I were used as the internal control. The primer sequences for Giop were as follows: Giop F: 5′-TCC TGC AAT TCG CAT TAC GT-3′, Giop R: 5′-GGT TGC AGC TAA TGG ATT CCT A-3′, and Giop P: 5′-6- FAM-TCG CAG TTC GCA GCG TTC A-None-3′ with the fluorescent reporter FAM (6-carboxyfluorescein). The primer sequences for Res were as follows: Res F: 5′-CGA GGA ATT TAG AAA CGC TCA AC-3′, Res R: 5′-GCT GTG CCA ATG GCT AGG A-3′, and Res P: 5′-HEX-TGC CAA GGG TCC GGC CAC C-BHQ2-3′.

##### 2.3.2.3 Singleplex and duplex reaction

Real-time PCR assays were conducted using an Applied Biosystems StepOnePlus™ System (Thermo Fisher Scientific Inc., Waltham, USA). The thermocyling conditions comprised an initial incubation at 95°C for 1 min, followed by 40 cycles of incubation 95°C for 15 s and 60°C for 30 s. Singleplex reaction mixtures contained 1× Luna Universal Probe qPCR Master Mix (New England Biolabs, Ipswich), 250 nM of probes (GiopP or ResP), 900 nM of the *P. viticola* primers (Giop F/R) or 120 nM of *V. vinifera* primers, and 2 μL of DNA template in a final volume of 10 μL. Duplex reaction mixtures contained 1× Luna Universal Probe qPCR Master Mix, 250 nM of both *P. viticola* and *V. vinifera* probes (Giop P and Res P), 900 nM of *P. viticola* primers (Giop F/R), 120 nM of *V. vinifera* primers (Res F/R), and 2 μL of DNA template in a final volume of 10 μL. Standard curves, DNA calibration, and qPCR optimization were done as previously described by Taibi et al. (2023). The molecular analysis from the present study and the one cited (Taibi et al., 2023) were performed at the same time by the same operator and using the same material and machinery.

##### 2.3.2.4 Molecular infestation index (MII)

The total DNA from four biological replicates of each sample was extracted as described in Subsection 2.3.2.1. Duplex qPCR assays were performed with three technical replicates of each template DNA. Water control and calibration DNA consisting in 10 ng/μL of *P. viticola* DNA were included for each assay. DNA levels were finally obtained by transforming the quantification cycles (Cq) values of both targets (*P. viticola* and *V. vinifera*) based on the standard curves previously derived by Taibi et al. (2023), as described below: DNA = 10^[(Cq − a)/b], where a = 21.899 and b = −3.4373 for *P. viticola* (in ng/μL), and a = 26.352 and b = −3.2958 for *V. vinifera* (in ng/μL).

*Pv* DNA in the presence of *V. vinifera* DNA in leaf samples was then expressed in terms of a molecular infestation index (MII), which was calculated as the ratio between the DNA concentrations of *P. viticola* and *V. vinifera* (Chu et al., 2019; Yan et al., 2012).

### 2.4 Assessment of oospore germination

Leaf litter samples (1 g per replicate of each leaf litter) were collected at weekly intervals from mid- March till the end of June of each year to check the germination course of oospores present in the five leaf litters. Oospore germination was assessed as previously described by Vercesi et al. (2010). The oospore suspension obtained from each leaf litter sample was used to inoculate three agar plates, serving as technical replicates, containing 1% water-agar (Agar Noble, Difco, Thermo Fisher Scientific); three 10-μl drops containing 100 oospores each were placed onto the agar of each plate. The plates were incubated at an optimal temperature of 20°C. Macrosporangium formation was monitored daily using a stereomicroscope (Leica Wild M10) from day 1 to day 16 post-incubation. The presence of the macrosporangium was used as an indicator of oospore germination; after observation, macrosporangia were removed using a syringe to prevent double counting. The number of germinated oospores was used to calculate the percentage of germinated oospores over the total number of oospores (i.e., 300 oospores per plate).

### 2.5 Assessment of DM severity on grapevine leaves

The whole canopy of each plant was carefully observed from bud break till the end of primary inoculum season (i.e., the season in which the inoculum generated by oospores contributes to epidemic development; Caffi et al., 2009), which was estimated using the epidemiological weather- driven model previously proposed by Rossi et al. (2008b). The main developmental stages of vines were assessed using the scale of Lorenz et al. (1995).

From the 1^st^ of April, all the leaves of each plant were enumerated and individually checked on a weekly basis. The number of leaves with DM lesions per plant and the number of lesions per leaf were recorded. The data obtained from the seven plants in a block were then averaged. All the leaves showing sign of infection by downy mildew were promptly removed to avoid any sporulation and possible secondary infections. Considering that the onset of new DM symptoms would be more likely in rainy periods, the observations were conducted twice a week in the periods after rain and once a week in the dry periods. Surrounding, DM-resistant varieties were also inspected in the eventuality of DM onset, and any affected leaves, if found, were removed.

### 2.6 Data analysis

An analysis of variance (ANOVA) was used to test the differences between years and leaf litters for the number of oospores per g of leaf litter, MII, number of DM-affected leaves per plant, and number of DM lesions per plant. Before ANOVA, the numbers were transformed using the natural logarithm function to obtain homogeneous variances. A *post-hoc* comparison of means was conducted using the Student-Newman-Keuls test at P < 0.05.

A bivariate correlation analysis was conducted by calculating the Pearson correlation coefficients between the abovementioned variables. In this analysis, another variable was introduced, that is, the number of (theoretically) germinating oospores, which was calculated by multiplying the number of oospores in the leaf litter by the average percentage of germinated oospores in a year.

The effect of leaf litter on DM onset was assessed through a survival analysis, a statistical method for censored time-to-event data (Lee and Wang, 2003). The non-parametric Kaplan-Meier analysis was used to measure the time for DM causing more than one lesion per plant starting from bud break. Weeks (hereinafter referred to as week of the year, WOY) following bud break were the time intervals for the analysis. The probability of becoming affected by DM during any of these intervals was calculated by dividing the leaf number that became affected by the leaf number at the start of the interval. The survival probability was plotted against time through stepped curves, with each step representing an event. The null hypothesis was that the probability of becoming affected is the same in all groups (LL0 to LL100). A pairwise comparison was made using the Breslow estimator.

All statistical analyses were carried out using SPSS (version 24; IBM SPSS Statistics, IBM Corp., USA).

## 3 Results

### 3.1 Oospores in leaf litters

The number of oospores in leaf fragments overwintered above the vineyard ground was significantly higher in 2023 (average of the five leaf litters: 4.72 × 10^5^ per g dry weight) than in 2021 (average: 6.25 × 10^4^ per g dry weight) and 2022 (average: 4.37 × 10^4^ per g dry weight) (P < 0.001). The year accounted for 74.2% of the experimental variance. The overall germination rate of oospores was very low. In 2021, the average germination in the bioassay was 0.30 ± 0.13%, with germination occurring in 45% of the samples collected during the season. The germination was lower in 2022 and 2023, being 0.01 ± 0.003% in both years, with germinated oospores being found in only 5.3% and 4.6% of seasonal samples, respectively.

**Figure 2:**
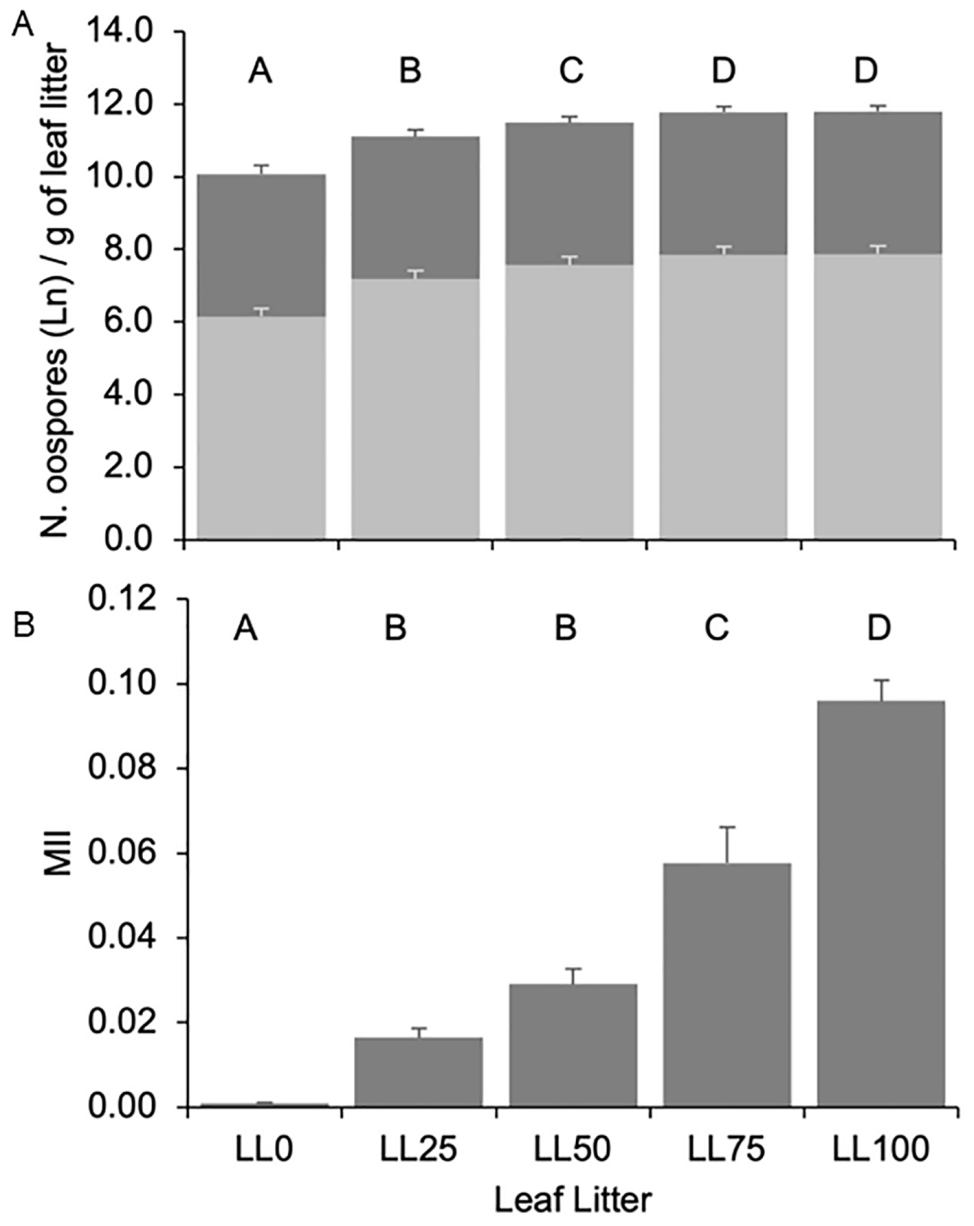
Number of total *ln*-transformed (dark grey bars, panel **A**) and germinating [light grey bars, panel **(A)**] *Plasmopara viticola* oospores, and molecular infestation index [MII, panel **(B)**] in the five grapevine leaf litters previously composed with the following proportions: 100% DM-free (LL0), 75% DM-free and 25% DM-affected (LL25), 50% DM-free and 50% DM-affected (LL50), 25% DM-free and 75% DM-affected (LL75), and 100 DM-affected (LL100). The five leaf litters were overwintered on the vineyard ground. Bars and whiskers represent the averages and standard errors, respectively. The letters above the bars show significant differences based on the Student–Newman– Keuls (S-N-K) test at P = 0.05. The numbers of oospores were counted with the help of a stereomicroscope and expressed as per g of leaf litter. The number of germinating oospores was calculated by multiplying the number of oospores by the average germination rate of the oospores in a year. MII was calculated as the ratio between the DNAs of *P. viticola* (in pg) and *Vitis vinifera* (in ng).

Oospore numbers increased with the proportion of DM-affected leaves in the leaf litter (P < 0.001) (**Figure 2A**). The oospore numbers in LL75 and LL100 were 1.25-fold higher than in LL25 and LL50 and 1.63-fold higher than in LL0. A number of oospores (9.19 × 10^4^ per g dry weight in the 3-year average) was also found in LL0, indicating the presence of resting spores in the leaves that did not show any visible DM symptoms in the field. The leaf litter and interaction leaf litter × year accounted for 22.1% and 3.7% of the variance, respectively.

As for the oospore numbers, the 3-year average of MII increased with the increasing proportion of DM-affected leaves in the leaf litter (P < 0.001). MII < 0.001 was found in LL0, which progressively increased to 0.096 in LL100 (**Figure 2B**). Both oospore numbers and MII significantly correlated with the percentage of DM-affected leaves in the leaf litter and to each other (**Table 1**), indicating that the two variables could be consistently used to estimate the oospore infestation in the DM-affected leaves and overwintered above the vineyard ground.

**Table 1:** Pearson correlation coefficients between the characteristics of the leaf litter (LL) above the vineyard ground, presence of *Plasmopara viticola* oospores, and downy mildew (DM) symptoms on plants.

	LL%	NOOS	NGER	MII	NLEA	NLES
% DM-affected leaves in leaf litter (LL%)	1^1^	0.969	0.984	0.974	0.981	0.991
		*0.007^2^*	*0.002*	*0.005*	*0.003*	*0.001*
Number of oospores in LL (NOOS)		1	0.996	0.895	0.911	0.941
			*<.0001*	*0.04*	*0.031*	*0.017*
Number of germinating oospores in LL (NGER)			1	0.929	0.933	0.967
				*0.022*	*0.021*	*0.007*
Molecular infestation index of LL (MII)				1	0.981	0.992
					*0.003*	*<0.001*
Number of leaves with DM lesions per plant (NLEA)					1	0.974
						*0.005*
Number of DM lesions per plant (NLES)						1

^1^Pearson's correlation coefficient

^2^p-value of Pearson correlation

### 3.2 Dynamics of DM on leaves

**Figure 3:**
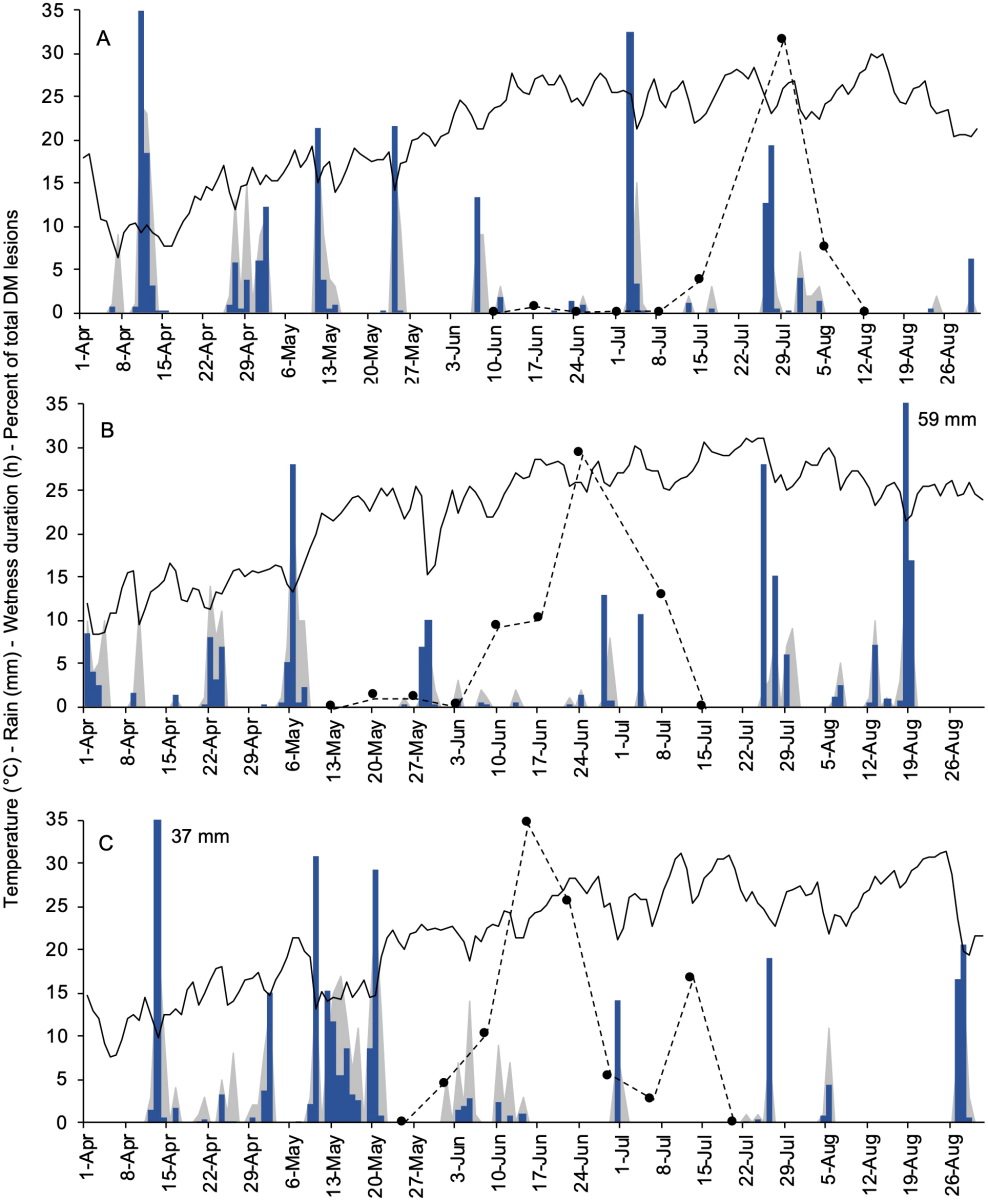
Weather data registered at the experimental site (University campus at Piacenza, Northern Italy) during the primary inoculum season of *Plasmopara viticola* in 2021 **(A)**, 2022 **(B)**, and 2023 **(C)**. Black lines, blue bars, and grey area depict the daily air temperature (°C), rainfall (mm in a day), and wetness duration (hours in a day), respectively. Black dots with dotted lines represent the percent of the total of new downy mildew lesions found on grapevine leaves cv. Merlot in a week.

In 2021 (**Figure 3A**), the plants were sprouting in early April, but no DM lesions were observed until mid-June. Most DM symptoms appeared late in the season, between mid-July and early August, following a total of 71 mm of rain that occurred on nine days between July 3 and the end of July. During that time, only the last 10% of the seasonal oospore dose was still available based on the model previously proposed by Rossi et al., 2008b (not shown). In 2022 (**Figure 3B**), bud break was observed during mid-April. First DM lesions were noticed on May 16, following repeated rainfall between May 4 and 8 (36 mm rain with a total of 52 wet hours). The majority of symptoms appeared in approximately one month, between early June and early July, mostly in the last ten days of June following weak but repeated rain events. In that period, more than 50% of the seasonal oospores were predicted by the model to break dormancy (not shown). In 2023 (**Figure 3C**), the first leaves were emerging in mid-April. The weather was very moist during the first three weeks of May (with a total of 138 mm of rain), which favored the development of most oospores (based on the model, not shown) and led to a repeated disease onset between late May and mid-June. A second period of DM onset was also recorded in the first ten days of July, following a single rain event with 14 mm of rain on June 30.

### 3.3 Effects of leaf litter on DM onset

**Figure 4:**
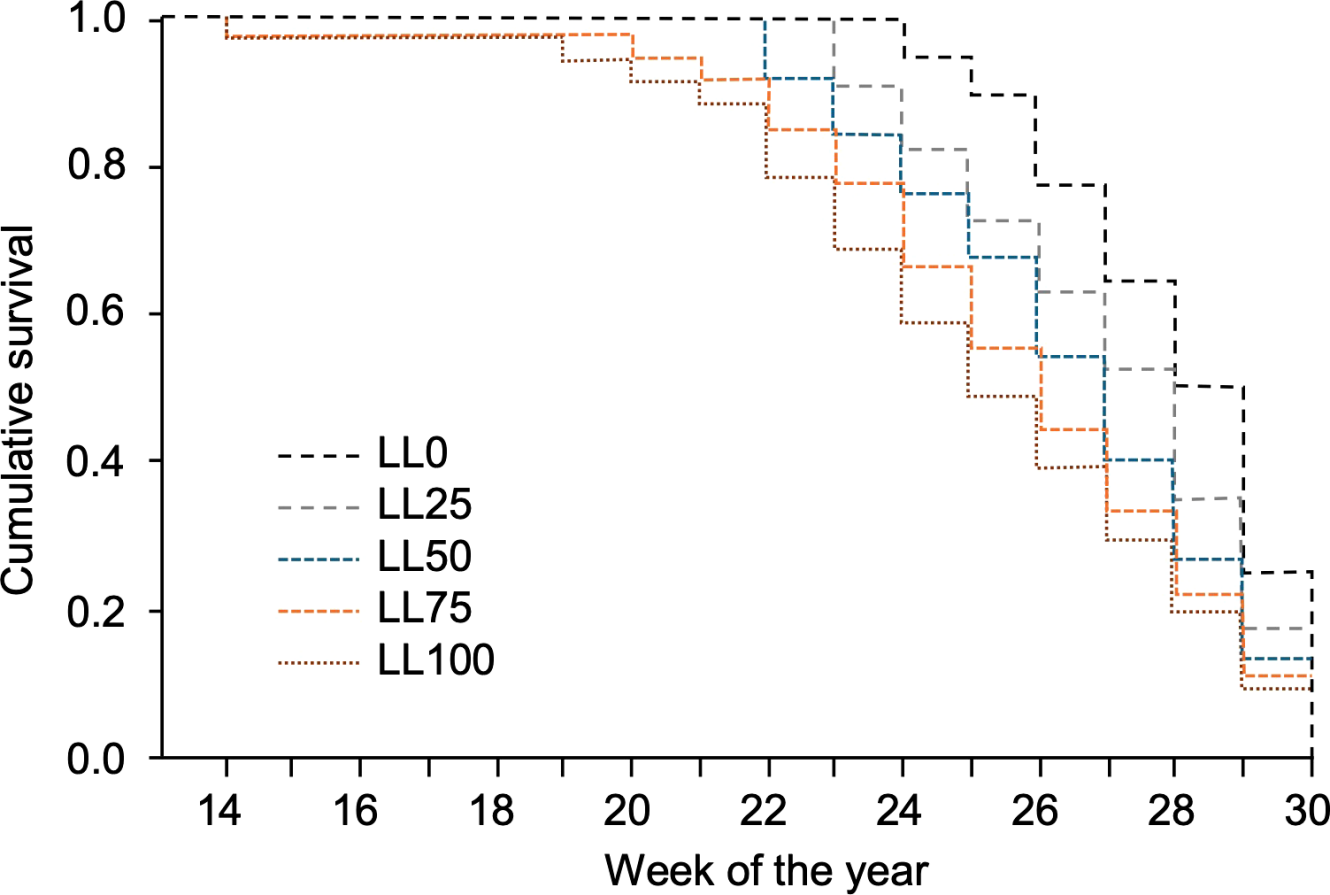
Survival functions representing the probability that no more than one grapevine downy mildew (DM) lesion per plant was detected on the plants grown in 2021–2023 on five grapevine leaf litters previously composed with the following proportions: 100% DM-free (LL0), 75% DM-free and 25% DM-affected (LL25), 50% DM-free and 50% DM-affected (LL50), 25% DM-free and 75% DM-affected (LL75), and 100 DM-affected (LL100). The five leaf litters were overwintered on the vineyard ground.

Survival analysis showed a significant impact of the leaf litter on the time of relevant DM onset (i.e., more than one affected leaf per plant) during the season (**Figure 4**). The overall comparison through the Breslow estimator gave χ^2^ = 8.32 (df = 1), which was significant with P = 0.004. The mean survival time progressively decreased from week 28 for LL0 to week 25.3 for LL100. Thus, the leaves in plants over LL50, LL75, and LL100 were affected 2.8, 1.9, and 1.3 weeks earlier than those over LL0 (**Table 2**).

**Table 2:** Survival time, expressed as weeks of the year (WOY) for more than one grapevine downy mildew (DM) lesion per plant to appear on plants grown between 2021 to 2023, on a soil covered with leaf litters previously composed with the following proportions: 100% DM-free (LL0), 75% DM-free and 25% DM-affected (LL25), 50% DM-free and 50% DM-affected (LL50), 25% DM-free and 75% DM-affected (LL75), and 100 DM-affected (LL100).

Leaf Litter	WOY	95% confidence interval	LL25		LL50		LL75		LL100	
			χ2	*P*	χ2	*P*	χ2	*P*	χ2	*P*
Overall (years 2021 to 2023)										
LL0	28.0	27.2 – 28.9	1.829	*0.176*	4.659	*0.031*	9.105	*0.003*	12.262	*<0.001*
LL25	27.2	26.2 – 28.2			0.778	*0.378*	4.018	*0.045*	6.367	*0.012*
LL50	26.6	25.6 – 27.6					1.542	*0.214*	3.152	*0.076*
LL75	25.7	24.6 – 26.8							0.261	*0.610*
LL100	25.3	24.2 – 26.4								
Year 2021										
LL0	29.5	28.5 – 30.5	0.628	*0.428*	2.602	*0.107*	4.109	*0.043*	6.182	*0.013*
LL25	29.0	27.9 – 30.1			1.334	*0.248*	3.499	*0.061*	5.232	*0.022*
LL50	28.0	26.6 – 29.4					1.933	*0.164*	2.798	*0.094*
LL75	26.2	24.1 – 28.4							0	*1*
LL100	26.0	24.2 – 27.8								
Year 2022										
LL0	27.0	25.4 – 28.6	0.205	*0.65*	0.736	*0.391*	2.354	*0.125*	4.284	*0.038*
LL25	26.5	24.8 – 28.2			0.181	*0.671*	1.345	*0.246*	3.08	*0.079*
LL50	26.0	24.2 – 27.8					0.596	*0.44*	2.009	*0.156*
LL75	25.0	23.0 – 27.0							0.499	*0.480*
LL100	24.0	21.9 – 26.1								
Year 2023										
LL0	28.0	26.6 – 29.4	1.833	*0.176*	2.798	*0.094*	2.798	*0.094*	2.798	*0.094*
LL25	26.5	24.8 – 28.2			0.181	*0.671*	0.181	*0.671*	0.181	*0.671*
LL50	26.0	24.2 – 27.8					0	*1*	0	*1*
LL75	26.0	24.2 – 27.8							0	*1*
LL100	26.0	24.2 – 27.8								

The five leaf litters were overwintered on the vineyard ground. χ^2^ refers to the Breslow estimator for pairwise comparisons between leaf litters, and *P* represents the probability of rejecting the null hypothesis, that is, the probability of becoming affected is the same for all leaf litters.

The differences between leaf litters were more evident in 2021 and 2022 than in 2023 (**Table 2**). The difference in DM onset between LL0 and LL100 was 3.5 weeks in 2021 (P = 0.013), 3 weeks in 2022 (P = 0.038), and 2 weeks in 2023 (P = 0.094). Overall, the differences among the leaf litters with different proportions of DM-affected leaves were less evident in 2023 than in previous years (**Table 2**). Since the oospore dose in leaf litters was higher in 2023 than in 2021 and 2022, the delay in DM onset for leaf litters with lower proportions of DM-affected leaves (LL0 and LL25) was more evident in samples with lower oospore numbers than those with higher oospore numbers.

### 3.4 Effects of leaf litter on DM severity

**Figure 5:**
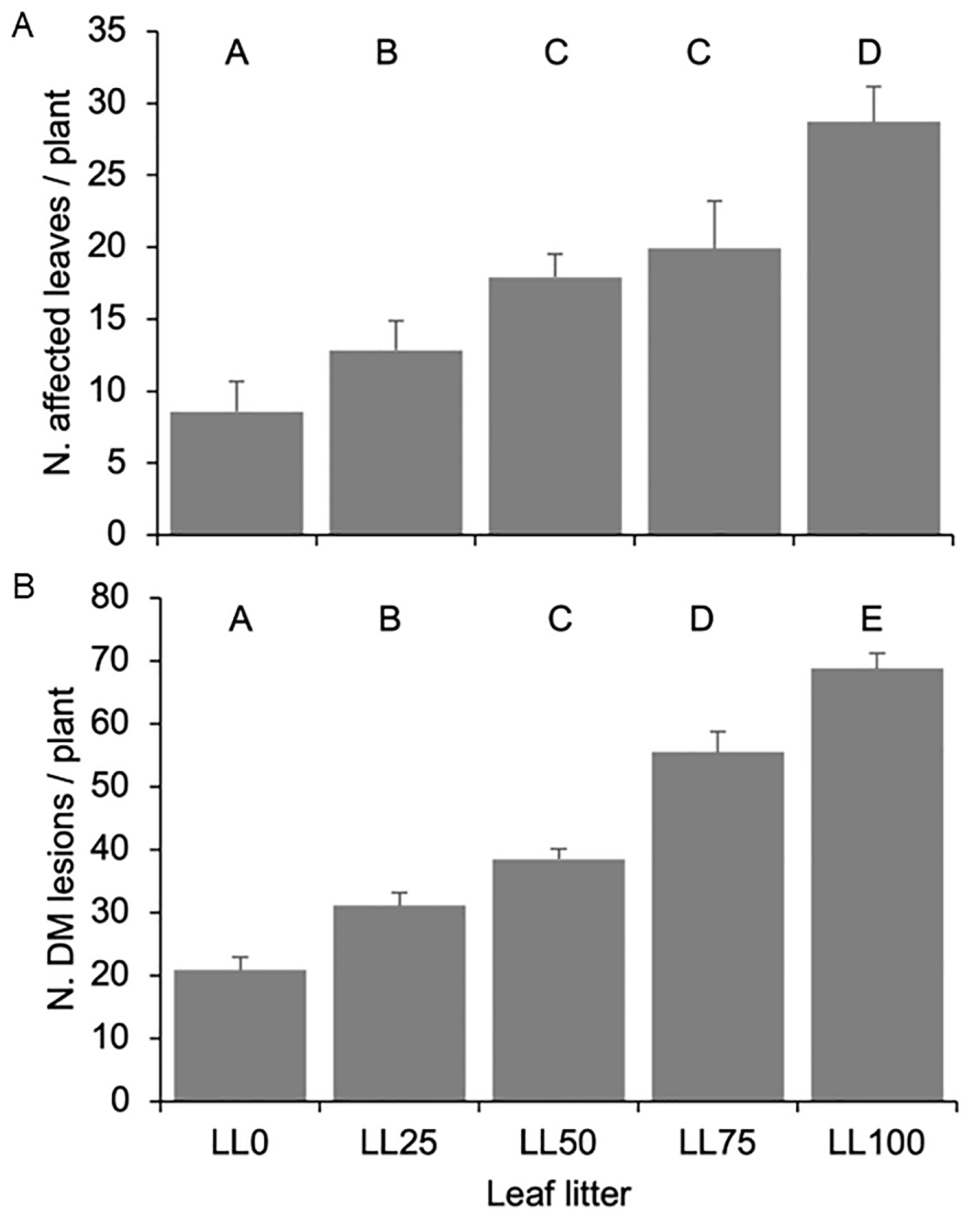
The number of leaves affected by *Plasmopara viticola* **(A)** and the number of downy mildew (DM) lesions on grapevine plants **(B)** grown on five leaf litters previously composed with the following proportions: 100% DM-free (LL0), 75% DM-free and 25% DM- affected (LL25), 50% DM-free and 50% DM-affected (LL50), 25% DM-free and 75% DM-affected (LL75), and 100 DM-affected (LL100). The five leaf litters were overwintered on the vineyard ground. Bars and whiskers represent the averages and standard errors, respectively. The letters above the bars show significant differences based on the Student–Newman–Keuls (S-N-K) test at P = 0.05.

The number of DM-affected leaves during the primary *P. viticola* inoculum season was significantly (P < 0.001) affected by the year (which accounted for 43.2% of experimental variance). Among the five leaf litters, an average of 11.6 ± 1.7, 11.5 ± 1.2, and 29.6 ± 1.8 of DM-affected leaves per plant were detected in 2021, 2023, and 2022, respectively. Considering that each plant had an average of 96 leaves at growth stage (GS) 65, the DM incidence was approximately 13–33%, depending on the year. The number of DM-affected leaves significantly increased from 8.6 ± 1.3 per plant above LL0 to 28.7 ± 5.7 per plant above LL100 (P < 0.001, **Figure 5A**). The leaf litter and interaction leaf litter × year accounted for 40.4% and 16.4% of the variance, respectively.

The number of DM lesions per plant followed a similar trend as the number of affected leaves. An average of 33.6 ± 4.3, 73.5 ± 4.7, and 21.8 ± 2.5 lesions per plant were observed in 2021, 2022, and 2023, respectively (P < 0.001), with the year accounting for 50.3% of the variance. The number of lesions increased with increasing proportion of DM-affected leaves in the leaf litter, with plants above LL100 exhibiting 4-fold more lesions than the plants above LL0 (P < 0.001, **Figure 5B**). The leaf litter and interaction leaf litter × year accounted for 30.2% and 19.5% of the variance, respectively. Interestingly, the average number of lesions per DM-affected leaf was quite constant across the different leaf litters (average: 2.44 ± 0.11, minimum: 2.15, and maximum: 2.79).

**Figure 6:**
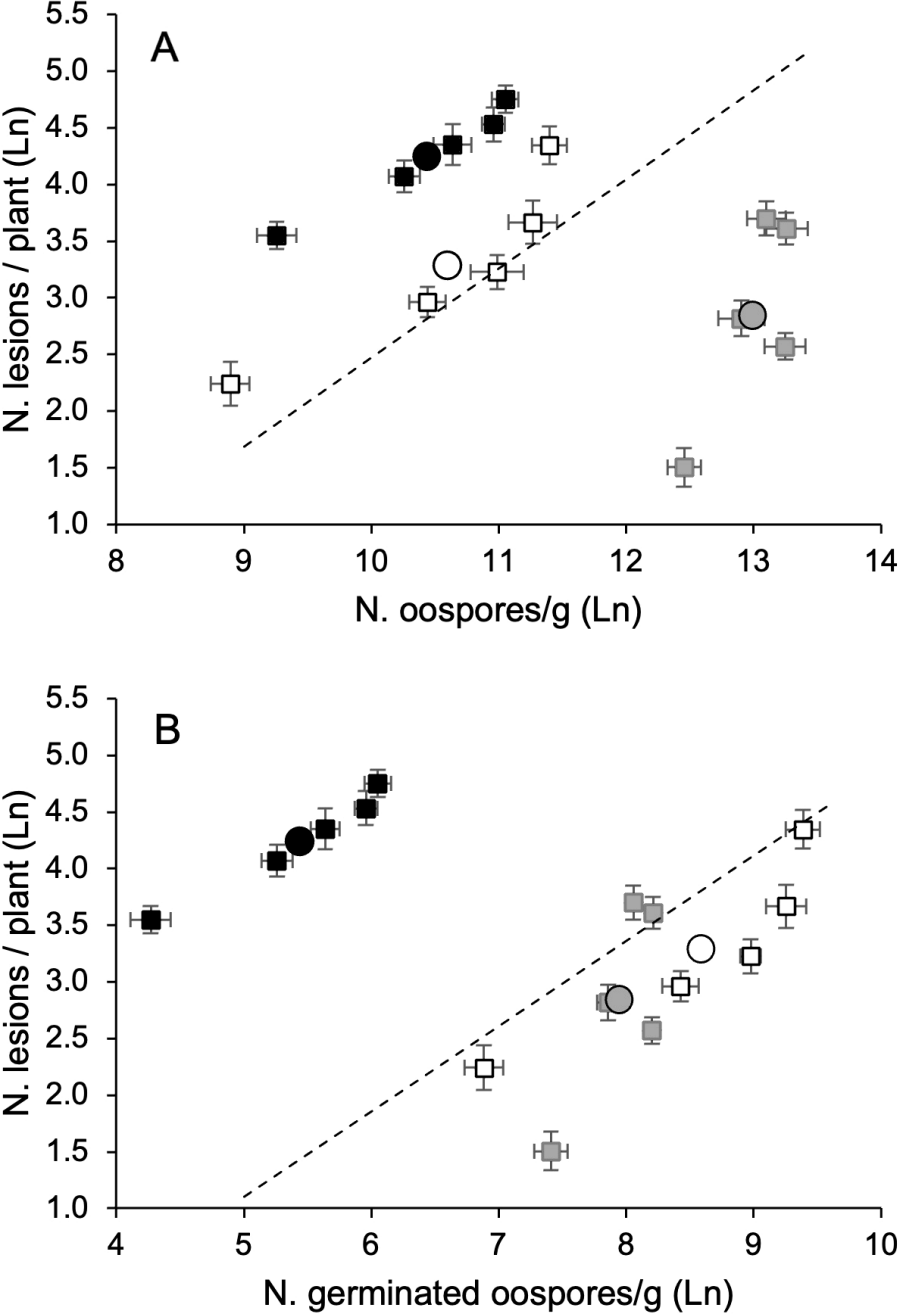
Relationship between the number of *Plasmopara viticola* oospores, either total **(A)** or germinating **(B)**, and numbers of downy mildew lesions on grapevisnes cv. Merlot, in 2021 (white dots), 2022 (black dots), and 2023 (grey dots). Whiskers represent the standard errors. In each year, the oospore numbers refer to the five leaf litters previously composed with the following proportions: 100% DM-free (LL0), 75% DM-free and 25% DM-affected (LL25), 50% DM-free and 50% DM- affected (LL50), 25% DM-free and 75% DM-affected (LL75), and 100 DM-affected (LL100). The five leaf litters were overwintered on the vineyard ground. The circles represent the average of the leaf litters each year. The dotted lines represent the linear regression lines (see text).

The average number of DM-affected leaves and lesions per plant significantly correlated with each other and with either the number of oospores in the leaf litter or MII (**Table 1**). The number of DM lesions per plant exhibited a higher correlation with the number of germinating oospores than with the total number of oospores in the leaf litter (0.967 vs. 0.941, **Table 1**), indicating that the oospore germination rate was relevant in determining the disease severity on the leaves. These relationships were linear because the quadratic component of polynomial regressions was not significant (not shown). For instance, the regression line (y = a + bx) for the natural logarithm of oospore (x) and DM lesion (y) numbers was significant (P = 0.012), with a = −5.386 (P = 0.044), b = 0.786 (P = 0.012), and R^2adj^ = 0.910 (**Figure 6A**). The regression was also significant for germinating oospores (P = 0.09), with a = −2.662 (P = 0.079), b = 0.753 (P = 0.009), and R^2adj^ = 0.924 (**Figure 6B**).

The analysis of this relationship (oospore numbers vs. DM lesions) over the three years revealed that the number of oospores in the leaf litter was not a good predictor of the number of DM lesions in plants. As already stated, the average number of oospores from all five leaf litters in 2023 was significantly higher than those in 2021 and 2022, but the number of DM lesions in plants was significantly higher in 2022 (73.5 per plant), than in 2021 (33.6 per plant) and 2023 (21.8 per plant) (P < 0.001, **Figure 6**). However, for each year, the number of lesions increased with the oospore numbers (total or germinated) in the leaf litter (**Figure 6**).

## 4 Discussion

This study aimed to investigate whether assessing the oospore pool in a vineyard before the start of the growing season can provide information about the potential DM pressure in the vineyard. This information could be used for informing disease management in vineyards. In our 3-year study, we found clear and close relationships between the percentage of grapevine leaves affected by *P. viticola* forming the leaf litter, the number of oospores in the leaf litter, and the development of primary infections on the leaves in the next season. We also found that the disease onset was delayed and the disease severity decreased with the reduction in the oospore pool.

Our results were consistent with the general dynamic of polycyclic epidemics, where a reduction in the primary inoculum led to a longer lag phase and a shift of the S-shaped (logistic) disease progress curve over time (Van der Plank, 1963). This ensures less infections in the early season when the vegetation is highly susceptible to DM (Wilcox et al., 2015) and, consequently, a slower build-up of the secondary inoculum so that late infections are synonymous with lower losses (Savary et al., 2009).

Our results were also in agreement with those of Carisse (2016). In eastern Canadian vineyards, plots with fall DM incidences of 0–2.5%, >2.5–5%, >5–10%, and >10–20% were selected and monitored in the following season from bud break until harvest. Fall disease incidence had a significant effect on DM progress: higher fall mildew incidence resulted in an earlier disease development and a higher disease severity at bloom in the following spring. Based on these results, Carisse (2016) postulated that when high numbers of leaves are infected in the fall, higher levels of primary inoculum (oospores) are present in the following spring. Our results were also in agreement with Yang et al. (2023), who found significant correlations between the number of oospores in leaf residue in the soil (estimated by a standard curve relating the quantity of *Pv* DNA and oospore numbers) and the *Pv* DNA in the grapevine leaves in commercial Chinese vineyards.

Unlike these studies, instead of considering natural infestation of vineyards, we created five leaf litters by mixing different proportions of fall DM-affected and (visually) healthy leaves to investigate a wider range of possible infestation levels and determine the differences among the different infestation levels. As expected, our 220 samples showed a wide range of oospore numbers, ranging from 2,940 to 783,000 per g of leaf litter. Data distribution was asymmetric with skewness = 1.256 and kurtosis = 0.139, with 50% of data in the range of 33,605–324,000 (**Supplementary Figure S1**). Carisse (2016) did not investigate the numbers of oospores in the affected fall leaves, and Yang et al. (2023) estimated the number of oospores per g of soil by extracting *Pv* DNA from the leaf residue in the soil samples. Hence, our data cannot be compared to these studies, making impossible the comparison between the levels of infestation created in this study and the ones in natural leaf litters. This finding, however, emphasizes the need for standardized protocols for the extraction of oospores or *Pv* DNA from overwintering sites, which would make the data comparison across studies possible.

We used three methods to assess the oospore pool in the leaf litters: (i) indirect estimation through the prevalence of DM-affected leaves in the leaf litter, similar to the method of Carisse (2016); (ii) indirect assessment through *Pv* DNA quantification, similar to the method of Yang et al. (2023); and (iii) direct microscopic counting of the oospores. The third method was employed to have a direct assessment of oospore numbers in our leaf litters. Among the different methods to directly count the oospore numbers in grapevine leaves, we used microscopic observations after filtration of the leaf tissue (Vercesi et al., 2000; Toffolatti et al., 2007), because methods based on leaf clearing and staining is time-consuming and is more suitable for green leaves (Burruano, 2000; Díez-Navajas et al., 2007; Taylor, 2018). The “floating grape leaf disc” bioassay - which comprises an indirect estimation of the oospore numbers via their germination in water and capability of infecting non-infested floating green leaf discs (Hill, 1998) - is more suited to studying the germination dynamics over the season than to assessing the oospore numbers (Lehoczky, 1956; Vercesi et al., 2002, 2010; Hill, 2001; Pertot and Zulini, 2003; Toffolatti et al., 2004; Rossi et al., 2008a; Caffi et al., 2009). To investigate the oospore germinability in our samples and then assess the potential of our oospore pool to cause an infection, we used the method of Vercesi et al. (2010), which is based on direct, microscopic observation of the formation of macrosporangia. This method is less time consuming than an indirect estimation through the floating leaf disc bioassay. The oospore germination rate in our samples was generally low, ranging from <1 to 1.2% (**Supplementary Figure S2**). Previous studies also showed that only a few of the oospores sampled during a season germinate (e.g., Burruano et al., 1989; Serra and Borgo, 1995; Toffolatti et al., 2006). For instance, in a previous 4-year study, the average oospore germination per sample was <3%, with 35% of samples exhibiting a rate of ≤1% (Maddalena et al., 2021). Such a low germination rate might be attributed to (i) dormancy duration, making oospores physiologically mature (e.g., able to germinate under favorable conditions) for months in a season (Gobbin et al., 2005; Kennelly et al., 2007; Caffi et al., 2011; Rossi et al., 2008b); (ii) oospore germinability, since oospores lose germinability over the years (Caffi et al., 2011); and (iii) the suppressive effect some fungi, yeasts, and bacteria colonizing the leaf litter (Vecchione et al., 2004).

*Pv* DNA was extracted from fallen leaves and quantified via qPCR using previously described methods to quantify *P. viticola* in soil and leaf residue (Si Ammour et al., 2020; Yang et al., 2023; Yuan, 2021). Using duplex qPCR, we then calculated an MII, which was similar to the molecular disease index already used for studying latent infections by pathogens (Yan et al., 2012; Chu et al., 2019; Luo et al., 2019), including *P. viticola* in grapevine (Yang et al., 2024). Oospore counting and *Pv* DNA quantification provided consistent results, being highly correlated with the proportion of DM-affected leaves in the leaf litter and with each other (r = 0.895). This finding confirmed that qPCR assay can be used for a rapid and robust assessment of the oospore pool in a vineyard.

Unexpectedly, both microscope counts and qPCR revealed the presence of oospores in our leaf litters with no DM-affected leaves (LL0). This finding could be attributed to an inaccurate estimation of the disease status of the leaves at the time of their collection in the vineyard, potentially due to their asymptomatic status, presence of light mosaic symptoms, and/or masking of the symptoms by color changes in senescing leaves. This finding indicated that the oospore dose in a vineyard cannot be accurately determined based on the incidence of DM-affected leaves.

Our study showed that the concept of epi-season, introduced by Magarey (2010) to describe the epidemiology of *Erysiphe necator*, can be applied to *P. viticola*, with some modifications. Based on this concept, the chasmothecia of *E. necator* develop in season 1, the first of two growing seasons in the “season of the epidemic” or “epi-season”. Chasmothecia are produced late in season 1, discharge ascospores early in season 2, and do not carry over to season 3. So, the powdery mildew epi-season covers a rolling window of two grapevine-growing seasons. Therefore, in season 1, *E. necator* produces an inoculum that survives over the winter as a legacy for season 2 when it triggers infection. This legacy factor in a vineyard is the main factor influencing the degree of the disease in early season and, thus, the level of difficulty in achieving good control of powdery mildew each season (Emmett and Magarey, 2008; Caffi et al., 2012, 2013). In the case of *P. viticola*, the legacy/inheritance factor spans more than two seasons, since, unlike *E. necator*, its resting structures survive over the first winter, being able to germinate at decreasing rates for at least 65 months (Caffi et al., 2011).

The epi-season concept entails that the regulation of DM epidemics in a season might start in the previous season(s) by preventing the formation of inoculum and/or by its suppression. *P. viticola* oospores form in the affected leaves late in the season (from July to leaf fall in temperate climates; Zachos, 1959), usually when the disease control with highly effective, synthetic fungicides has stopped because ontogenic resistance of bunches makes infection risk nil or irrelevant. Indeed, in this period, the disease can only spread on the susceptible leaves of growing lateral shoots; its effect is not relevant to the current season yield, apart from severe cases of defoliation. Moreover, fungicides are not applied after harvesting. As a consequence, grapevines remain almost unprotected against DM for the whole period when oospores develop.

Prevention of oospore formation should then start from late-season control of DM. However, the data on the efficacy of fungicides in preventing oospore development is scarce (Bissbort et al., 1997; Wong and Wilcox, 2001; Vercesi et al., 2002). For instance, azoxystrobin applied twice at the end of August and mid-September reduced the numbers of oospores and their germinability, decreasing the inoculum potential by >90% (Vercesi et al., 2002). One might dispute that the use of fungicides in the late season would increase costs, impact the environment and human health, and increase the risk of fungicide resistance (Mueller et al., 2021). However, a careful evaluation is warranted in the frame of the epi-season concept, which is the balance between the increased defense in the fall and the reduced defense (or losses) in the next spring. The epi-season 2022–2023 in Italy was an interesting case study for this aspect. DM was very mild during the grapevine-growing season in 2022, with no or occasional produce losses. However, the disease spread on the leaves in the late season because of mild and wet weather, and oospores were produced abundantly (as we observed in the leaf samples we collected for forming the leaf litters of this work). In the spring of 2023, intensive and frequent rainfall in the early season (April and May) led to devastating epidemics with an almost complete loss of the developing clusters in several grapevine-growing areas, that were not stopped even with intensive applications of synthetic fungicides (Caffi and Furiosi, 2024). A similar situation occurred in France, where an emergency fund was created to assist the vine growers whose vineyards were severely affected by DM in 2023 (https://www.bignonlebray.com/en/20-million-euros-aid-for-vintners-affected-by-downy-mildew/).

Suppression of the oospores that have produced and entered into the vineyard oospore pool could be obtained by using microorganisms able to affect viability and prevent oospore germination. However, the research on effective biocontrol products against oospores is still ongoing (Vecchione et al., 2004; Dagostin et al., 2006; Burruano et al., 2016), and, to the best of our knowledge, no commercial products are available so far.

Overall, our research showed that qPCR can be used to assess the potential oospore pool in a vineyard; this would require an appropriate sampling plan of leaf litter in the vineyard, which needs to be developed. Knowledge about the oospore pool can be used for DM management in the early season, combined with the mathematical models able to predict whether the environmental conditions favor primary infection (Rossi et al., 2008b). With a high oospore pool, the disease should be carefully managed starting from the first susceptible growth stages of grapevines, while first seasonal model alarms could be disregarded in case of a very low oospore pool. The oospore pool size data could also be useful for soil management. For instance, in vineyards with a high oospore pool, specific cover crops could be sown that drastically limit the rain splashing from the soil to the vegetation, so limiting inoculum dispersal (Rossi and Caffi, 2012), delaying DM onset and reducing its severity (Hasanaliyeva et al., 2024).

The present study focused on the oospores that overwintered in the leaf litter above the vineyard ground, where their concentration is generally higher than in the soil (Yang et al., 2023). However, we can postulate that when the leaf litter decomposes with time (Nikolaidou et al., 2010), oospores remain in the leaf fragments (or leaf residue) incorporated into the soil and later become free in the soil. Thus, the overall oospore pool in a vineyard can be split into three components: (i) the leaf litter above the ground, composed of fallen leaves, which accumulate on the ground as a natural layer (Johnson and Catley, 2002); (ii) the leaf residue mixed in the soil after physical fragmentation and microbial decomposition (Tennakoon et al., 2021); and (iii) the soil itself. Oospores in these compartments might be different in numbers and age and experience different environmental/microbial conditions, impacting their viability and germination rate. For instance, the germination ability of *Peronospora viciae* oospores declined rapidly after incorporation in the soil (Van der Gaag, 1997).

The contribution of each of the three components (leaf litter, leaf residue, and soil) to the overall oospore pool of a vineyard is still poorly understood. Previously, Yang et al. (2023) buried the grapevines at a soil depth of 20–40 cm after harvest to protect them from frost damage and collected the soil samples containing the leaf residue from a depth of 10 cm below the roots in early April. Leaf residues were then carefully separated from the soil, and the number of oospores was estimated in the two fractions. The oospores were present in all the soil samples and in >80% of leaf residue samples. The oospore concentration was approximately 28-fold higher in the leaf residue than in the soil. A comprehensive study on the contribution of the abovementioned components to the overall oospore pool in vineyards and on the germination potential of these oospores is warranted to devise an effective strategy for leaf residue and soil management in vineyards.

## Data Availability

The raw data supporting the conclusions of this article will be made available by the authors, without undue reservation.
