# Supplementary material for: Relationship between the oospore dose in the leaf litter above the vineyard ground and primary infections by Plasmopara viticola
Source: Front Plant Sci. 2025 Mar 7;16:1524959. doi: 10.3389/fpls.2025.1524959 (PMC11925907; doi:10.3389/fpls.2025.1524959)
Supplement: Supplementary file 1 [file DataSheet1.docx]

Supplementary Material

# Supplementary Figures

#

**Supplementary Figure 1.** Variability in the numbers of *Plasmopara viticola* oospores found in 220 samples of grapevine leaf litter over three years (2021–2023).

**Supplementary Figure 2.** Variability in the germination frequency of *Plasmopara viticola* oospores **(A)** and the time required for oospore germination **(B)** in 220 samples of grapevine leaf litter over three years (2021–2023).
